# Changes in Sarcopenia Status and Subsequent Cardiovascular Outcomes: Prospective Cohort Study

**DOI:** 10.2196/69860

**Published:** 2025-09-08

**Authors:** Yuanyue Zhu, Kan Wang, Zuolin Lu, Feika Li, Yu Xu, Linhui Shen, Yufang Bi, Weiguo Hu

**Affiliations:** 1 Department of Geriatrics Ruijin Hospital, Shanghai Jiao Tong University School of Medicine Shanghai China; 2 Medical Center on Aging Ruijin Hospital, Shanghai Jiao Tong University School of Medicine Shanghai China; 3 Department of Endocrine and Metabolic Diseases, Shanghai Institute of Endocrine and Metabolic Diseases Ruijin Hospital, Shanghai Jiao Tong University School of Medicine Shanghai China; 4 National Clinical Research Center for Metabolic Diseases (Shanghai), Key Laboratory for Endocrine and Metabolic Diseases of the National Health Commission, National Research Center for Translational Medicine, State Key Laboratory of Medical Genomics Ruijin Hospital, Shanghai Jiao Tong University School of Medicine Shanghai China; 5 School of Population Medicine and Public Health Chinese Academy of Medical Sciences & Peking Union Medical College Beijing China

**Keywords:** sarcopenia, transition, air pollution, cardiovascular disease

## Abstract

**Background:**

Sarcopenia is associated with cardiovascular diseases (CVDs). However, whether changes in sarcopenia status affect CVD risk remains unclear. In addition, how indoor fuel use impacts the sarcopenia transition process is less well studied.

**Objective:**

This study prospectively examined the association of sarcopenia transitions with CVD risk, while exploring the effect of indoor fuel on these transitions.

**Methods:**

In this prospective observational study, we used data from the China Health and Retirement Longitudinal Study waves 1 to 4 (2011 to 2018). In total, 8739 participants with complete data on sarcopenia and indoor fuel use were included for the indoor fuel use and sarcopenia transition analysis, and 6385 participants without previous CVDs were included for the sarcopenia transition and CVD risk analysis. Sarcopenia transition was defined according to the sarcopenia status at wave 1 (2011) and wave 2 (2013). Incident CVDs included heart diseases, stroke, and composite CVDs. Information on indoor fuel use was obtained at wave 1. Cox proportional hazards models were used to examine the effect of sarcopenia transition on incident CVDs. Logistic regression models were used to investigate the impact of indoor fuel use on these transitions.

**Results:**

During a median of 7.0 years of follow-up, 1233 incident CVDs were documented. Compared to stably normal participants, progressing from a normal state to possible or confirmed sarcopenia brought increased risk of incident CVD (hazard ratio 1.42, 95% CI 1.15-1.77). Conversely, recovering to a normal state was associated with decreased risk (hazard ratio 0.72, 95% CI 0.55-0.95) for baseline participants with possible sarcopenia. In addition, clean fuel use increased the odds of achieving a possible-to-normal transformation (odds ratio 1.32, 95% CI 1.06-1.64), while both solid cooking and heating fuel use were associated with a higher risk of deterioration in sarcopenia status.

**Conclusions:**

An unfavorable transition in sarcopenia status is associated with higher CVD risk, while reversion from possible sarcopenia to a normal state could reduce the risk. Therefore, early intervention for sarcopenia is imperative for CVD prevention, and promoting clean indoor fuel use is recommended.

## Introduction

### Background

Sarcopenia is a common age-related condition, characterized by the loss of skeletal muscle mass, strength, and decline in physical function [1]. The prevalence of sarcopenia has been rising worldwide, with an estimated prevalence of 10% to 16% among older adults [2]. Globally, the epidemic of sarcopenia continuously imposes considerable health care and economic burdens as populations age.

Recent studies have shown that the clinical implications of sarcopenia extend well beyond the musculoskeletal system. Instead, it is also closely associated with several other adverse outcomes, including a higher risk of hospitalization and premature mortality [3,4]. Furthermore, emerging evidence suggests that sarcopenia is significantly associated with cardiovascular disease (CVD) [5,6]. For example, Gao et al [6] found that both possible and diagnosed sarcopenia were associated with higher CVD risk. This finding is further supported by an analysis of National Health and Nutrition Examination Survey, which demonstrated that sarcopenia served as an independent risk factor for CVD [7]. In addition, Mendelian randomization studies demonstrated a genetic correlation between sarcopenia and CVD development [8]. Indeed, sarcopenia and CVD have many shared risk factors, including insulin resistance, poor nutrition, smoking, and sedentary lifestyle [9,10]. Mechanistically, the loss of muscle mass can lead to chronic inflammation and insulin resistance, both established contributors to cardiovascular pathogenesis [11]. Moreover, imbalance of anabolic and catabolic homeostasis with reduction of muscle loss might lead to cardiovascular deconditioning as well [12].

However, much of the existing literature focused on the effect of sarcopenia status on CVD at single time points, with less attention paid to the changes in sarcopenia stages during follow-up [7,13-15]. In fact, sarcopenia is not a static condition; rather, it follows a dynamic progression. Starting from a subtle decline of muscle mass, it will either subsequently progress to clinical sarcopenia with noticeable reductions in muscle strength and physical performance, be reversed to a more normal state, or be stabilized with consistent muscle mass and quality [16]. Revealing the longitudinal association of transition in sarcopenia status with CVD can provide a more comprehensive understanding of the effect of sarcopenia on cardiovascular health; more importantly, such insights may identify opportunities to reverse or delay the progression of CVD brought on by muscle disorders [17].

Since the change in sarcopenia status might have long-term effects on CVD, identifying predictors of certain transition stereotypes is clinically important for developing targeted interventions. Various environmental, lifestyle, and socioeconomic factors may impact the progression of sarcopenia, thereby indirectly affecting cardiovascular outcomes [18-20]. Among these potential determinants, indoor air pollution represents a particularly relevant exposure given that older individuals spend a majority of time indoors [21]. Considering the cross-sectionally reported association between indoor air pollution and sarcopenia [22,23], we hypothesize that it may also affect the transition of sarcopenia stages. Despite the potential connections, limited research has examined how indoor air pollution influences sarcopenia transitions and their cardiovascular consequences, representing a critical knowledge gap for clinical practice.

### Objectives

To fill these knowledge gaps, we aimed to explore the effect of sarcopenia transitions on the risk of subsequent CVDs and to figure out the role of indoor air pollution in specific changing patterns. Given the tremendous disease burden caused by sarcopenia and CVD, identifying modifiable environmental risk factors for sarcopenia progression and understanding their cardiovascular implications may contribute to more targeted intervention strategies.

## Methods

### Ethical Considerations

All study procedures have been approved by the Peking University Institutional Review Board (IRB00001052-11015). Participants provided written consent after being fully informed of the design and potential consequences of the study. To ensure privacy and confidentiality, no identifiable information has been included.

### Study Population

The China Health and Retirement Longitudinal Study (CHARLS) is a community-based, prospective survey of the Chinese population aged 45 years or above. The detailed study design has been described elsewhere [24]. Briefly, the baseline survey was conducted between June 2011 and March 2012 with individuals selected through multistage probability sampling. Follow-up is performed every 2 years with physical measurements and blood testing. Three follow-up waves (2013, 2015, and 2018) were completed after the baseline survey.

A detailed flowchart for participants’ selection in this study is shown in Figure S1 in Multimedia Appendix 1. Among 17,708 participants, 8969 (50.64 %) were excluded due to being younger than 45 years (n=777, 4.4 %) or having missing information on sarcopenia status at wave 1 in 2011 (n=3992, 22.54 %) or wave 2 in 2013 (n=4200, 23.72 %), leaving 8739 participants for the indoor fuel use and sarcopenia transition analysis. Then, 2354 (13.29 %) participants were further excluded due to prevalent CVDs before wave 2 (2013) or loss to follow-up afterward, leaving 6385 (36.06 %) participants included for sarcopenia transition and CVD risk analysis.

### Measurements and Definitions of Covariates

On the basis of a face-to-face computer-assisted personal interview system, structured questionnaires were administered and on-site physical measurements were conducted by trained field workers [25]. Information on socioeconomic factors (education level, marital status, household income, and urban and rural residence), lifestyle factors (drinking and smoking habits, sleep duration, and physical activity), and medical history (prevalent heart disease, stroke, diabetes, hypertension, chronic lung disease, asthma, and cancer, as well as antihypertensive and antidiabetic drugs) was collected through questionnaires. BMI was calculated as body weight divided by the square of height (kg/m^2^). Ideal physical activity level was defined as participating in moderate or vigorous activity more than once a week [26]. Ideal sleep duration was defined as having a sleep duration of 6 to 8 hours per night [27]. Mini-Mental State Examination (MMSE) scores were used to assess the cognitive functions [28].

### Measurement of Indoor Fuel Use

The CHARLS separately collects data on the energy source for cooking and heating purposes. Types of indoor fuel use were categorized according to a previous study [22]. Specifically, solid fuel was defined as the primary use of coal, crop residue, and wood. Clean fuel was defined as the primary use of solar energy, natural gas, liquefied petroleum gas, and electricity. This binary classification (solid vs clean) was consistently applied to both cooking fuel and heating fuel [29].

### Assessment of Sarcopenia Status

Sarcopenia status at wave 1 (2011) and wave 2 (2013) was assessed based on the criteria recommended by the Asian Working Group for Sarcopenia 2019 [30], including muscle strength, physical performance, and appendicular skeletal muscle mass (ASM). Handgrip strength (in kg) was measured by a standardized dynamometer (Yuejian WL-1000); the cut-off point for low handgrip strength was defined as less than 28 kg for men and less than 18 kg for women. ASM was estimated by a validated anthropometric equation, which showed strong agreement with dual x-ray absorptiometry [31,32], and the cut-off value for defining low muscle mass was based on the sex-specific lowest 20% of the height-adjusted muscle mass (ASM/height^2^) among the study population. Physical performance was estimated using the gait speed and the chair stand test. For gait speed, the time taken by each participant in walking a 2.5-m distance was recorded. The 5-time chair stand test measured the amount of time needed for the participants to rise continuously 5 times, keeping their arms folded across their chest, from the height of the 47-cm chair, and low physical performance was defined as a gait speed <1.0 m/s or a 5-time chair stand test ≥12 seconds [7].

According to the Asian Working Group for Sarcopenia 2019, possible sarcopenia is defined as low muscle strength or low physical performance, and confirmed sarcopenia is defined as low muscle mass plus low muscle strength or low physical performance. Therefore, all participants in this study were divided into 3 groups at each wave: normal, possible sarcopenia, and confirmed sarcopenia.

### Categorization of Sarcopenia Transitions

Transition in sarcopenia status was defined as that between waves 2011 and 2013. In total, 9 distinct transition patterns in sarcopenia were identified based on participants’ conditions in 2011 and 2013. To ensure clinical relevance, certain categories were combined. Specifically, the transitions from “normal” to “possible sarcopenia” and from “normal” to “confirmed sarcopenia” were grouped, as both reflect a decline from a healthy baseline and indicate any deterioration in muscle status. Similarly, the transition from “confirmed sarcopenia” to “normal or possible sarcopenia” was combined, as it represents an improvement from the most severe baseline state. Other categories were retained separately due to the distinctive clinical trajectories, despite smaller sample sizes.

### Ascertainment of CVDs

The primary outcome was composite CVDs, including heart disease and stroke. Similar to previous studies, CVDs were ascertained by the following questions in each wave: “Have you been told by a physician that you have been diagnosed with a heart attack, angina, coronary heart disease, heart failure, or other heart problems?” “Have you been told by a doctor that you have been diagnosed with a stroke?” Participants who reported having heart disease or stroke were considered to have CVD [7,33]. In subsequent survey waves, participants were asked to confirm the existence of heart disease or stroke reported in the past wave. Once they disputed their earlier reports, the diagnosis would be retrospectively corrected [34]. Participants were followed until the first incident of CVD, death, or the end of the study period (the wave in 2018), whichever came first.

### Statistical Analysis

Baseline characteristics are presented as mean (SD) for normally distributed continuous variables, median (IQR) for nonnormally distributed continuous variables, and number (percentage) for categorical variables. For continuous variables, 1-way ANOVA (for normally distributed variables) or the Kruskal-Wallis test (for nonnormally distributed variables) was used to test for overall differences across groups. For categorical variables, chi-square tests were used. The Sankey diagram was constructed to indicate the transitions of sarcopenia between 2011 and 2013.

Cox proportional hazard models were used to estimate the hazard ratios (HRs) and 95% CIs for incident CVDs associated with sarcopenia transitions. Adjustments included baseline age; sex; BMI; marital status (yes or no); high school education (yes or no); rural residence (yes or no); household income; smoking status (never, former, or current smoker); drinking status (never, former, or current drinker); optimal physical activity (yes or no); ideal sleep duration (yes or no); antihypertensive and antidiabetic medication; chronic diseases (including diabetes, hypertension, chronic lung disease, asthma, and cancer); MMSE scores; and types of fuel use (solid or clean). The proportional hazard assumption was verified through the visual inspection of scaled Schoenfeld residual plots, with no significant deviations observed. In addition, the main analyses were repeated using specific cardiovascular outcomes (heart disease and stroke). To examine the association between baseline fuel use and sarcopenia status transitions between waves 2011 and 2013, multivariable logistic regression models were used. All models were adjusted for the aforementioned covariates except for the types of fuel use.

In addition, we performed stratified analyses according to age group (aged ≥65 y vs <65 y), sex, smoking status, physical activity level, and diabetes status to assess potential effect modification. These subgroup variables were selected based on their established associations with both sarcopenia and cardiovascular risk [5]. Statistical significance of multiplicative interaction was evaluated by the likelihood-ratio test [35]. The missing data of covariates were imputed using the multiple imputation with chained equations method [36]. On the basis of a previous study, only those with missing rates less than 20% would be imputed, multiple imputations are set to 10 times, not at least 10 times [37]. Effect estimates were computed separately for these datasets and then combined according to the Rubin rules [38]. To assess the robustness of our findings, we performed sensitivity analyses restricted to observations with complete covariate information. Data were handled and analyzed with SPSS statistics (version 25.0.0.1; IBM Corp) [39] and R CRAN (version 4.3.2; R Foundation) [40]. Specifically, we used the *networkD3* package of R software to generate the Sankey visualizations. All analyses were performed at a significance level of.05 (2 tailed).

## Results

### Baseline Characteristics

**Figure 1:**
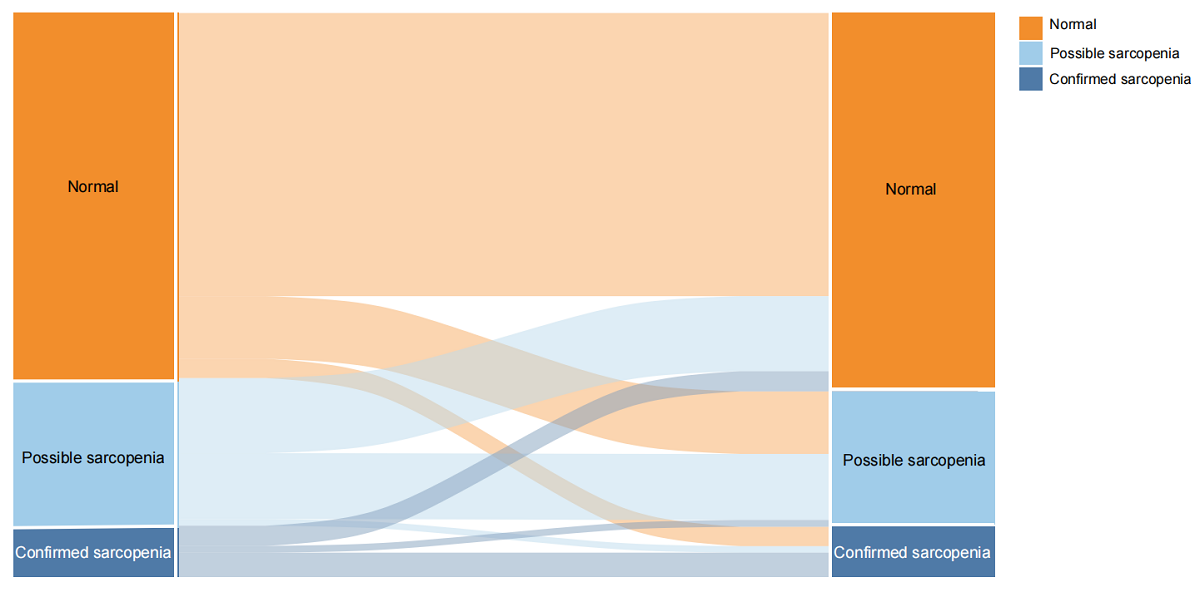
Transition of sarcopenia status between wave 1 (2011) and wave 2 (2013) in the China Health and Retirement Longitudinal Study (CHARLS).

For the analysis of sarcopenia transition and CVD risk, the distribution and the exact numbers of various transition patterns between wave 1 (2011) and wave 2 (2013) is shown in Figure 1.

Baseline characteristics varied significantly across sarcopenia transition groups (Table 1). The stable normal group demonstrated the most advantaged socioeconomic profiles, being the youngest (mean age 56.2, SD 7.7 years), with the highest proportion having high school education (n=397, 11.8%), the greatest likelihood of being married (n=3134, 93.00%), and the highest income levels (median CN ¥14,770, IQR ¥3367.5-¥37,082.5; The exchange rate of US dollars to Chinese yuan is US $1=CN ¥7.15), along with the highest MMSE scores (median 16.0, IQR 13.0-19.0) and greatest representation in rural areas (n=577, 17.1%). In contrast, the stable confirmed sarcopenia group comprised the oldest participants (mean age 70.9, SD 8.4 years) of whom the rates of high school education, marriage, and income levels were also the lowest. Meanwhile, the stable possible sarcopenia group displayed the most concerning lifestyle and health profiles, with the highest prevalence of being a current smoker (n=419, 66.1%), current drinker (n=428, 67.3%), prevalent diabetes (n=476, 74.6%), prevalent hypertension (n=345, 54.1%), and the incidence of heart disease (n=139, 21.9%) and stroke (n=73, 11.6%). Notably, individuals transitioning from possible to confirmed sarcopenia presented with the poorest cognitive and physical function, evidenced by the lowest MMSE scores (median 11.0, IQR 7.0-15.5) and the lowest rate of ideal physical activity levels (n=32, 49%).

Regarding indoor fuel use and sarcopenia transition analysis, baseline characteristics between different transition paradigms were shown in Table S1 in Multimedia Appendix 1, with similar patterns observed.

**Table 1:** Baseline characteristics of the participants for sarcopenia transition and cardiovascular disease analysis.

			Stable normal (n=3370)		Normal to possible or confirmed sarcopenia (n=890)		Possible sarcopenia to normal (n=827)		Stable possible sarcopenia (n=637)		Possible to confirmed sarcopenia (n=66)		Confirmed sarcopenia to normal or possible (n=297)		Stable confirmed sarcopenia (n=298)		*P* value	
Age (y), mean (SD)			56.2 (7.7)		59. 7 (8.7)		57.3 (7.5)		62.3 (9.1)		67.4 (9.8)		64.7 (8.6)		70.9 (8.4)		<.001	
Men, n (%)			1788 (53.1)		405 (45.5)		359 (43.4)		251 (39.4)		37 (56.1)		134 (45.1)		112 (37.6)		<.001	
High school or above, n (%)			397 (11.8)		50 (5.6)		61 (7.4)		23 (3.6)		1 (1.5)		12 (4)		7 (2.3)		<.001	
Married, n (%)			3134 (93.0)		774 (87)		746 (90.2)		533 (83.7)		46 (69.7)		241 (81.1)		196 (65.8)		<.001	
Income (CN ¥^a^)			14,770.0 (3367.5-37,082.5)		9900.0 (2460.0-28,950.0)		9100.0 (2360.0-30,000.0)		6200.0 (1325.0-26,875.0)		6240.0 (725.0-21,700.0)		4630.0 (1494.8-18,150.0)		3400.0 (900.0-18,122.5)		<.001	
Rural, n (%)			577 (17.1)		90 (10.1)		121 (14.6)		81 (12.7)		4 (6.1)		19 (6.4)		23 (7.7)		<.001	
BMI (kg/m^2^), mean (SD)			23.6 (3.8)		22.9 (3.7)		24.2 (3.0)		24.9 (3.5)		22.3 (3.6)		19.1 (1.6)		19.1 (1.9)		<.001	
MMSE^b^			16.0 (13.0-19.0)		14.0 (11.0-17.0)		15.0 (12.0-18.0)		13.0 (10.0-17.0)		11.0 (7.0-15.5)		14.0 (11.0-17.0)		12.0 (9.0-14.0)		<.001	
**Smoking status, n (%)**																		<.001
	Never	1165 (34.6)		269 (30.2)		242 (29.3)		165 (25.9)		24 (36.4)		94 (31.6)		91 (30.5)				
	Former	267 (7.9)		71 (8)		62 (7.5)		50 (7.8)		3 (4.5)		16 (5.4)		15 (5)				
	Current	1935 (57.4)		548 (61.6)		518 (62.6)		419 (65.8)		38 (57.6)		186 (62.6)		191 (64.1)				
**Drinking status, n (%)**																		<.001
	Never	1292 (38.3)		291 (32.7)		256 (31)		151 (23.7)		17 (25.8)		97 (32.7)		69 (23.2)				
	Former	247 (7.3)		55 (6.2)		68 (8.2)		57 (8.9)		7 (10.6)		25 (8.4)		36 (12.1)				
	Current	1829 (54.3)		544 (61.1)		502 (60.7)		428 (67.2)		41 (62.1)		174 (58.6)		194 (65.1)				
Ideal physical activity, n (%)			2693 (79.9)		667 (74.9)		589 (71.2)		400 (62.8)		32 (48.5)		220 (74.1)		170 (57)		<.001	
Optimal sleep duration, n (%)			2234 (66.3)		527 (59.2)		527 (63.7)		371 (58.2)		34 (51.5)		134 (45.1)		124 (41.6)		<.001	
**Chronic diseases, n (%)**																		
	Hypertension	1076 (31.9)		342 (38.4)		287 (34.7)		345 (54.2)		22 (33.3)		90 (30.3)		128 (43)		<.001		
	Diabetes	2405 (71.4)		626 (70.3)		600 (72.6)		476 (74.7)		45 (68.2)		211 (71)		210 (70.5)		0.006		
	Chronic lung disease	239 (7.1)		81 (9.1)		66 (8)		66 (10.4)		7 (10.6)		32 (10.8)		40 (13.4)		0.001		
	Asthma	96 (2.8)		41 (4.6)		27 (3.3)		33 (5.2)		4 (6.1)		15 (5.1)		20 (6.7)		0.001		
	Cancer	22 (0.7)		5 (0.6)		7 (0.8)		8 (1.3)		0 (0)		1 (0.3)		0 (0)		0.345		
	Incident stroke	146 (4.3)		48 (5.4)		50 (6)		73 (11.5)		5 (7.6)		19 (6.4)		20 (6.7)				
	Incident heart disease	369 (10.9)		144 (16.2)		112 (13.5)		139 (21.8)		13 (19.7)		40 (13.5)		55 (18.5)				
Antihypertensive drugs, n (%)			402 (11.9)		126 (14.2)		128 (15.5)		182 (28.6)		8 (12.1)		26 (8.8)		43 (14.4)		<.001	
Antidiabetic drugs, n (%)			83 (2.5)		22 (2.5)		26 (3.1)		31 (4.9)		2 (3)		5 (1.7)		3 (1)		0.008	
Solid fuel for cooking, n (%)			1850 (54.9)		587 (66)		533 (64.4)		443 (69.5)		51 (77.3)		216 (72.7)		234 (78.5)		<.001	
Solid fuel for heating, n (%)			2598 (77.1)		745 (83.7)		691 (83.6)		541 (84.9)		62 (93.9)		268 (90.2)		265 (88.9)		<.001	
Handgrip strength, n (%)			35.0 (29.0-42.8)		31.5 (26.5-38.0)		30.0 (24.0-37.3)		27.0 (21.0-34.5)		25.5 (21.6-30.3)		26.0 (20.5-33.0)		23.0 (17.1-27.0)		<.001	
Appendicular skeletal muscle (kg)			17.8 (14.3-20.8)		16.4 (13.4-19.6)		16.9 (14.2-20.2)		16.7 (13.9-19.9)		17.1 (12.2-18.6)		12.3 (10.5-17.3)		11.4 (10.1-16.1)		<.001	

^a^US $1=CN ¥7.15.

^b^MMSE: Mini-Mental State Examination.

### Association Between Transition in Sarcopenia Status and CVD

**Figure 2:**
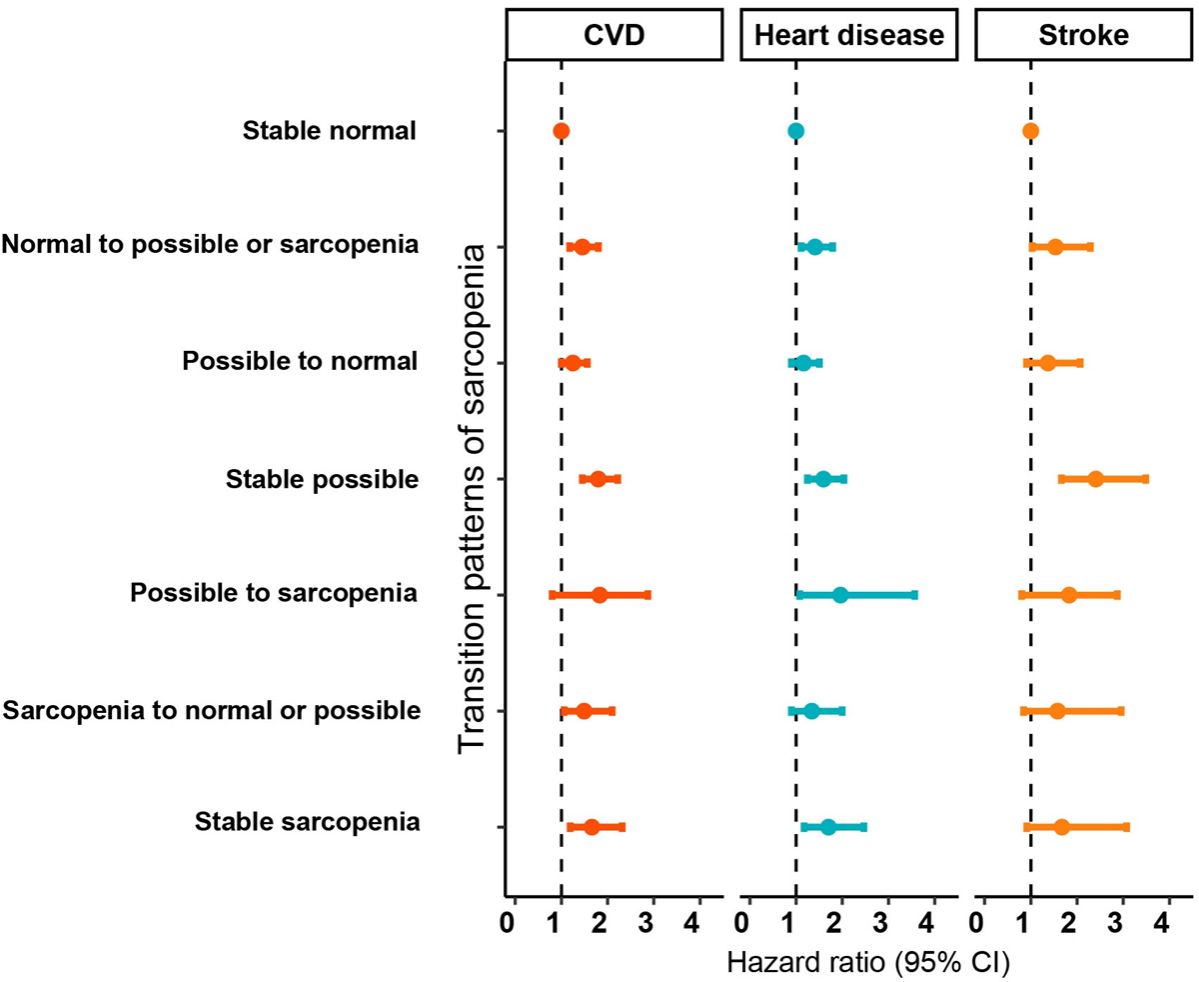
Associations between sarcopenia transitions and the risk of cardiovascular diseases.

The effect of transition in sarcopenia status on CVD risk during follow-up is shown in Figure 2. Participants maintaining stably normal muscle function harbored the lowest risk for composite CVD and also its subtypes and were thus used as the reference.

Compared with the reference group, HRs (95% CIs) for those who stayed possible sarcopenia and confirmed sarcopenia were 1.71 (1.43-2.05) and 1.55 (1.16-2.06), respectively. For participants undergoing an unfavorable shift to a more severe state, significantly higher risks of CVD were also observed. Specifically, progression from normal to possible or confirmed sarcopenia and possible to confirmed sarcopenia were associated with a 40% and 73% higher risk of CVD (Table S2 in Multimedia Appendix 1) compared with those maintaining a normal state, respectively. Importantly, the risk of CVD was still elevated among patients recovering from confirmed to possible sarcopenia and a normal state (HR 1.42, 95% CI 1.05-1.90). In contrast, no significant increase in CVD risk was observed in the “possible sarcopenia to normal” group (HR 1.13, 95% CI 0.94-1.36). Results were generally consistent for heart diseases. However, the risk of stroke was only significantly elevated among stable possible (HR 2.04, 95% CI 1.51-2.76). Further stratification analysis revealed that the association between sarcopenia transition and CVD was more prominent among male, physically active, and nondiabetic individuals (Tables S3-S7 in Multimedia Appendix 1).

Because most participants maintained their baseline status during follow-up, we subsequently used 3 stable groups as the corresponding references (eg, those who remained in a normal state, those with possible sarcopenia, and those with confirmed sarcopenia throughout the study period). Associations between sarcopenia transition and CVD of different baseline sarcopenia statuses are shown in Table 2. Among participants with normal baseline status, those who progressed to possible or confirmed sarcopenia showed elevated risk of CVD (HR 1.42, 95% CI 1.15-1.77) compared to stable normal participants. For those with possible sarcopenia at baseline, participants who improved to normal status demonstrated reduced risk (HR 0.72, 95% CI 0.55-0.95), while those who progressed to confirmed sarcopenia showed potentially higher risk, although without statistical significance. Moreover, compared with participants maintaining confirmed sarcopenia at baseline, those who recovered to possible sarcopenia or normal status had an HR of 0.80 (95% CI 0.51-1.23). Regarding specific outcomes, deterioration from normal status at baseline was associated with a 40% increased risk of heart disease, while the remission from possible sarcopenia to a normal state was not associated with significantly elevated risk. Moreover, recovery from possible sarcopenia to a normal state was associated with a lower risk of stroke (HR 0.54, 95% CI 0.34-0.86), compared with their stable counterparts. Results of stratification analysis within different baseline sarcopenia statuses were presented in Tables S8 to S12 in Multimedia Appendix 1, and significant interaction effects of sex and physical activity were observed.

**Table 2:** Associations between sarcopenia transition and cardiovascular diseases of different baseline sarcopenia statuses.

Sarcopenia transition	Cardiovascular disease, HR^a^ (95% CI)	Heart disease, HR (95% CI)	Stroke, HR (95% CI)
Stable normal	1.00 (reference)	1.00 (reference)	1.00 (reference)
Normal to possible or confirmed sarcopenia	1.42 (1.15-1.77)	1.40 (1.10-1.79)	1.42 (0.94-2.14)
Stable possible sarcopenia	1.00 (reference)	1.00 (reference)	1.00 (reference)
Possible sarcopenia to normal	0.72 (0.55-0.95)	0.80 (0.58-1.00)	0.54 (0.34-0.86)
Possible to confirmed sarcopenia	1.09 (0.62-1.93)	1.16 (0.61-2.22)	1.05 (0.29-2.38)
Stable confirmed sarcopenia	1.00 (reference)	1.00 (reference)	1.00 (reference)
Confirmed sarcopenia to normal or possible	0.80 (0.51-1.23)	0.68 (0.41-1.12)	0.84 (0.37-1.90)

^a^HR: hazard ratio. Adjusted for baseline age, sex, BMI, married (yes or no); high school education (yes or no); rural residence (yes or no); household income; smoking status (never, former, or current smoker); drinking status (never, former, or current drinker); optimal physical activity (yes or no); ideal sleep duration (yes or no); antihypertensive and antidiabetic medication; prevalent diseases (including diabetes, hypertension, chronic lung disease, asthma, and cancer); and MMSE scores and types of fuel use (solid or clean).

### Associations of Types of Cooking and Heating Fuel Use With Sarcopenia Transitions

The effect of types of fuel use on transition is shown in Table 3. Briefly, clean fuel use is a prediction of improvement in sarcopenia status. Compared with solid fuel, clean fuel use increased the odds of achieving the possible-to-normal transformation by 32% (*P*=.01). Meanwhile, both solid heating fuel (odds ratio [OR] 1.32, 95% CI 1.14-1.53) and solid cooking fuel (OR 1.20, 95% CI 1.01-1.44) were associated with a higher risk of the progression from normal to possible or confirmed sarcopenia. The association between fuel use and confirmed sarcopenia to normal or possible sarcopenia was not significant. In addition, significant effect modification was observed for sex, physical activity, and diabetes status (Tables S13-S17 in Multimedia Appendix 1).

**Table 3:** Associations of the use of cooking and heating fuel and sarcopenia transition.

Fuel type	Normal to possible or confirmed sarcopenia			Possible sarcopenia to normal				Confirmed sarcopenia to normal or possible		
	Clean fuel, OR^a^ (95% CI)	Solid fuel, OR (95% CI)	*P* value	Clean fuel, OR (95% CI)	Solid fuel, OR (95% CI)	*P* value	Clean fuel, OR (95% CI)		Solid fuel, OR (95% CI)	*P* value
Cooking	1.00 (reference)	1.32 (1.14-1.53)	<.001	1.00 (reference)	1.32 (1.06-1.64)	.01	1.00 (reference)		1.16 (0.79-1.70)	.44
Heating	1.00 (reference)	1.20 (1.01-1.44)	.04	1.00 (reference)	0.92 (0.70-1.21)	.55	1.00 (reference)		0.98 (0.58-1.66)	.95

^a^OR: odds ratio.

Adjusted for baseline age, sex, BMI, married (yes or no); high school education (yes or no); rural residence (yes or no); household income; smoking status (never, former, or current smoker); drinking status (never, former, or current drinker); optimal physical activity (yes or no); ideal sleep duration (yes or no); antihypertensive and antidiabetic medication; prevalent diseases (including diabetes, hypertension, chronic lung disease, asthma, and cancer); and MMSE scores.

### Sensitivity Analysis

All missing rates of the covariates were <5% (Table S18 in Multimedia Appendix 1). Therefore, we repeated the analysis using the complete dataset without any missing covariates (Tables S19-S21 in Multimedia Appendix 1). These sensitivity analyses produced results consistent with the primary findings.

## Discussion

### Principal Findings

In this community-based cohort study conducted in China, we found that progression to worse sarcopenia status was associated with increased cardiovascular risk. Conversely, participants with possible sarcopenia at baseline but who returned to normal status demonstrated considerably reduced risk. Moreover, clean fuel use was associated with favorable transitions in sarcopenia status, whereas solid fuel use was a risk factor for negative shifts toward a deteriorated sarcopenia status.

Previous studies have well illustrated the association between different sarcopenia stages and CVD [5,7,14,15,41]. Despite these important observations, it is noteworthy that sarcopenia is a dynamic process rather than a stable state, and divergent changing paths of sarcopenia are associated with significantly distinct health outcomes [16]. For example, the transition toward deteriorated muscle mass was associated with metabolic abnormalities [42]. Unfortunately, little research has examined the association between sarcopenia transition and CVD risk.

In this study, we comprehensively reported that different trajectories of sarcopenia transitions were associated with markedly different risks of composite CVD. It is expected that a stably pathological state (ie, stable possible and confirmed sarcopenia) and the worsening in sarcopenia status were both associated with a higher risk of CVD. As shown in Table 1, the prevalence of hypertension and diabetes and the suboptimal lifestyle factor are higher among these categories. These negative influencers are also common risk factors of CVD. Moreover, reversion from possible sarcopenia to a normal state, but not from confirmed to possible sarcopenia, was associated with reduced risk. It is indicated that advanced sarcopenia involves increasingly irreversible metabolic alterations that persist despite apparent muscle mass recovery [43]. These findings suggest that intervention at the relatively early stages of sarcopenia may be more clinically beneficial. The weaker associations with stroke across all transition patterns may also reflect the mechanistic complexity of cerebrovascular disease, suggesting that the cardiovascular effects of sarcopenia may be more specific to cardiac rather than cerebrovascular pathways [7,9]. Methodological considerations also significantly contribute to our null findings. For example, the transition from possible to confirmed sarcopenia showed consistently nonsignificant associations across all cardiovascular outcomes, likely reflecting insufficient statistical power given the small sample size (n=66, 1 %), although point estimates generally favored increased risk. Similarly, the substantially reduced statistical power due to fewer stroke events (n=361, 5.7 %) compared to heart disease cases (n=872, 13.6 %) fundamentally limits our ability to detect modest but potentially clinically meaningful associations, emphasizing the need for larger studies with extended follow-up periods. We also noticed that the associations between sarcopenia transition and CVD were particularly pronounced in men, physically active individuals, and those without a history of diabetes. Given that both diabetes and physical inactivity are important risk factors for CVD, it may obscure or diminish the incremental risk associated with sarcopenia transition. Moreover, the gender difference in the effect of sarcopenia has been reported previously [44], which indicated that a male participant could exhibit a more pronounced susceptibility to the detrimental effects of sarcopenia.

Given that the transition and trajectory of sarcopenia have substantially varied health effects, understanding the risk factors is essential for developing effective interventions. Traditional determinants included aging, malnutrition, and physical inactivity [18,45]. However, little attention has been paid toward the environmental drivers. Previous studies indicated that fuel use was significantly associated with a higher risk of developing sarcopenia among the general population [46,47]. Our findings further demonstrated that solid fuel use for both cooking and heating contributed to declining muscle performance.

The mass and function of skeletal muscle progressively decline with aging, and sarcopenia is thus advancing [48]. This process is insidious and often accompanied by physical disability and chronic disease, including CVD. Sarcopenia is associated with cardiovascular remodeling and dysfunction through several pathways: insulin resistance, endothelial inflammation, oxidative stress, and neurohormonal abnormalities [49]. Efforts aimed at these pathways may help improve cardiovascular outcomes in patients with sarcopenia. Furthermore, explanations of how fuel use impacts the transition process remained uncertain. The hypothesis included harmful materials and heavy metals released by solid fuel, which induce neuromuscular degeneration [50,51]. Nonetheless, the exact mechanisms are yet to be explored.

### Strengths and Limitations

This study has several strengths. This prospective study provides important evidence on the effect of sarcopenia transition on CVD risk, with the longitudinal design minimizing potential reverse causality. In addition, most attention in sarcopenia research has been paid toward the older adults, despite that the decline in muscle mass and quality already begins in the early 40s [52]. By including participants aged 45 years and above, this provided insights into a more comprehensive scope. However, some limitations are to be addressed. First, CVDs were identified based on self-reported physician diagnosis. However, the validity of self-reported CVDs has been confirmed by previous studies using the CHARLS database [53]. Second, ASM values were estimated by an equation rather than dual x-ray absorptiometry or magnetic resonance, despite the validity of this method having been established in previous studies [7,16]. Third, the lack of detailed temporal fuel exposure data constrains our ability to examine the dose-response relationships and critical exposure windows for sarcopenia development. Finally, it is important to note that the CHARLS only consisted of Chinese participants, restricting the generalizability of our findings.

Our study suggested that the unfavorable transition of sarcopenia status is associated with a higher CVD risk, and reversion from possible sarcopenia may help reduce the related risk. Therefore, intervention at an earlier stage is imperative, and promoting the use of clean fuel may be helpful in averting muscle loss.

## Appendix

Multimedia Appendix 1 Sensitivity analysis of the association between sarcopenia transition, indoor fuel use, and cardiovascular diseases.

## Abbreviations

ASM: appendicular skeletal muscle mass
CHARLS: China Health and Retirement Longitudinal Study
CVD: cardiovascular disease
HR: hazard ratio
MMSE: Mini-Mental State Examination
OR: odds ratio

## Data Availability

The original China Health and Retirement Longitudinal Study dataset is available on the web. The full dataset used in this analysis is available from the corresponding author upon reasonable request.
