# Correction: Changes in Sarcopenia Status and Subsequent Cardiovascular Outcomes: Prospective Cohort Study

**DOI:** 10.2196/84642

**Published:** 2025-10-17

**Authors:** Yuanyue Zhu, Kan Wang, Zuolin Lu, Feika Li, Yu Xu, Linhui Shen, Yufang Bi, Weiguo Hu

**Affiliations:** 1 Department of Geriatrics Ruijin Hospital, Shanghai Jiao Tong University School of Medicine Shanghai China; 2 Medical Center on Aging, Ruijin Hospital, Shanghai Jiao Tong University School of Medicine Shanghai China; 3 Department of Endocrine and Metabolic Diseases, Shanghai Institute of Endocrine and Metabolic Diseases Ruijin Hospital, Shanghai Jiao Tong University School of Medicine Shanghai China; 4 National Clinical Research Center for Metabolic Diseases (Shanghai), Key Laboratory for Endocrine and Metabolic Diseases of the National Health Commission, National Research Center for Translational Medicine, State Key Laboratory of Medical Genomics Ruijin Hospital, Shanghai Jiao Tong University School of Medicine Shanghai China; 5 School of Population Medicine and Public Health, Chinese Academy of Medical Sciences & Peking Union Medical College Beijing China

In “Changes in Sarcopenia Status and Subsequent Cardiovascular Outcomes: Prospective Cohort Study” (JMIR Aging 2025;8:e69860) the authors noted one error.

In the originally published manuscript, the first author’s name appeared as “Yuanyuan Zhu” and their ORCID ID appeared as “0009-0007-4341-9468”.

The author’s name has been corrected to read as “Yuanyue Zhu”, and the ORCID ID was corrected to “0009-0001-6704-7036”.

The correction will appear in the online version of the paper on the JMIR Publications website, together with the publication of this correction notice. Because this was made after submission to PubMed, PubMed Central, and other full-text repositories, the corrected article has also been resubmitted to those repositories.

