# Supplementary material for: Changes in Sarcopenia Status and Subsequent Cardiovascular Outcomes: Prospective Cohort Study
Source: JMIR Aging. 2025 Sep 8;8:e69860. doi: 10.2196/69860 (PMC12455154; doi:10.2196/69860)
Supplement: Multimedia Appendix 1 [file aging_v8i1e69860_app1.docx]

**Contents**

**Figure S1.** Flowchart of the study

**Table S1.** Baseline characteristics of participants for indoor fuel use and sarcopenia transition analysis

**Table S2**. Associations between sarcopenia transitions and risk of cardiovascular diseases

**Table S3**. Associations between sarcopenia transitions and risk of cardiovascular diseases stratified by sex

**Table S4**. Associations between sarcopenia transitions and risk of cardiovascular diseases stratified by age group

**Table S5**. Associations between sarcopenia transitions and risk of cardiovascular diseases stratified by smoking status

**Table S6**. Associations between sarcopenia transitions and risk of cardiovascular diseases stratified by physical activity status

**Table S7**. Associations between sarcopenia transitions and risk of cardiovascular diseases stratified by diabetes status

**Table S8**. Associations between sarcopenia transitions and cardiovascular diseases of different baseline sarcopenia status stratified by sex

**Table S9**. Associations between sarcopenia transitions and cardiovascular diseases of different baseline sarcopenia status stratified by age group

**Table S10**. Associations between sarcopenia transitions and cardiovascular diseases of different baseline sarcopenia status stratified by smoking status

**Table S11**. Associations between sarcopenia transitions and cardiovascular diseases of different baseline sarcopenia status stratified by physical activity status

**Table S12**. Associations between sarcopenia transitions and cardiovascular diseases of different baseline sarcopenia status stratified by diabetes status

**Table S13.** Associations between types of indoor fuel use and sarcopenia transitions stratified by sex

**Table S14.** Associations between types of indoor fuel use and sarcopenia transitions stratified by age group

**Table S15.** Associations between types of indoor fuel use and sarcopenia transitions stratified by smoking status

**Table S16.** Associations between types of indoor fuel use and sarcopenia transitions stratified by physical activity status

**Table S17.** Associations between types of indoor fuel use and sarcopenia transitions stratified by diabetes status

**Table S18.** Missing rates of included variables

**Table S19**. Associations between sarcopenia transitions and risk of cardiovascular diseases (missing covariates deleted)

**Table S20**. Associations between sarcopenia transitions and cardiovascular diseases of different baseline sarcopenia status (missing covariates deleted)

**Table S21**. Associations between types of indoor fuel use and sarcopenia transitions (missing covariates deleted)


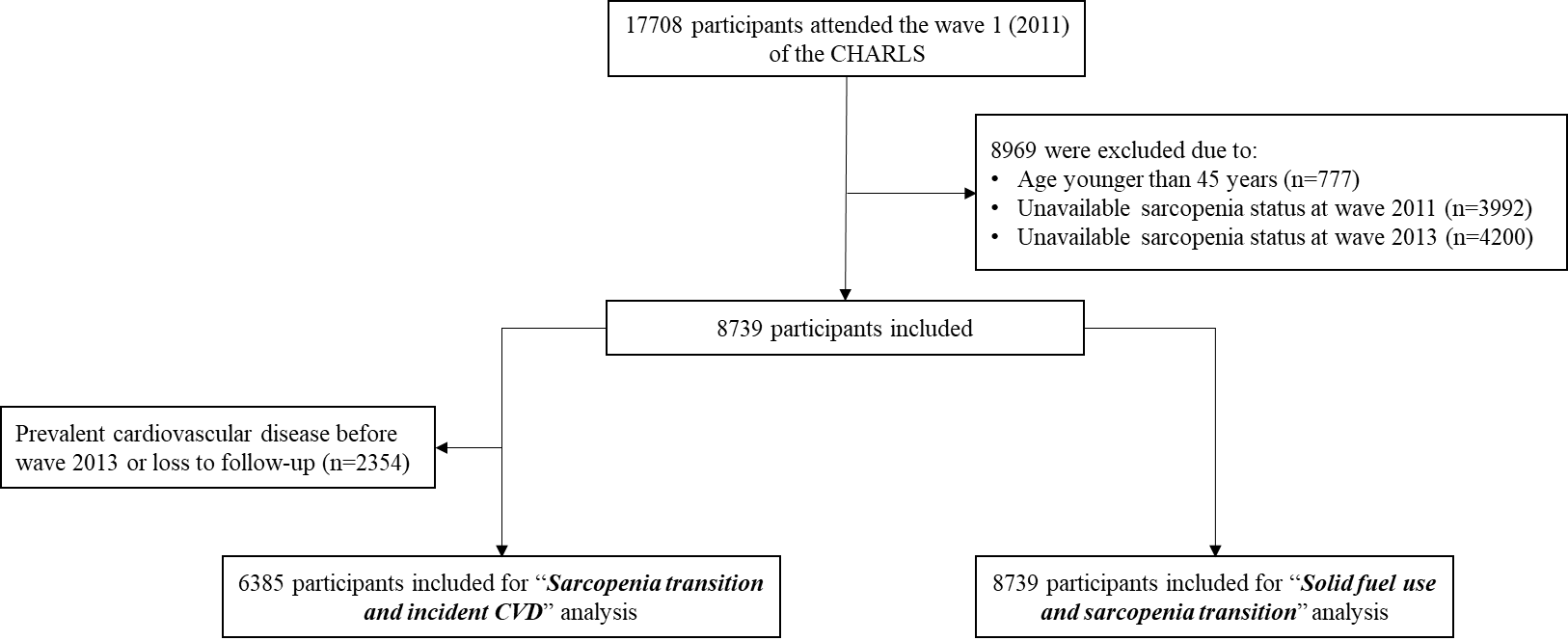
Figure S1

**Table S1.** Baseline characteristics of participants for indoor fuel use and sarcopenia transition analysis

|  | **Stable normal** | **Normal to**  **Possible/confirmed sarcopenia** | **Possible sarcopenia**  **to normal** | **Stable possible sarcopenia** | **Possible to**  **confirmed sarcopenia** | **Confirmed sarcopenia**  **to normal/possible** | **Stable confirmed**  **sarcopenia** | **P for trend** |
| --- | --- | --- | --- | --- | --- | --- | --- | --- |
|  | 4367 | 1252 | 1143 | 1027 | 95 | 394 | 461 |  |
| Age at baseline, years | 56.4±7.8 | 60.1±8.9 | 57.8±7.8 | 62.5±8.9 | 67.8±9.5 | 65.1±8.6 | 71.5±8.4 | <0.001 |
| Men, % | 2283 (52.3) | 572 (45.7) | 465 (40.7) | 392 (38.2) | 46 (48.4) | 174 (44.2) | 187 (40.6) | <0.001 |
| High school or above, % | 580 (13.3) | 98 (7.8) | 93 (8.1) | 51 (5.0) | 2 (2.1) | 15 (3.8) | 11 (2.4) | <0.001 |
| Married, % | 4028 (92.2) | 1088 (86.9) | 1030 (90.1) | 850 (82.8) | 69 (72.6) | 310 (78.7) | 300 (65.1) | <0.001 |
| Income | 15000.0  [3207.0, 37200.0] | 10975.0  [2770.0, 31105.0] | 10000.0  [2400.0, 30060.0] | 6876.0  [1500.0, 27720.0] | 4775.0  [940.0, 20925.0] | 4490.0  [1300.0, 16945.0] | 3580.0  [840.0, 20550.0] | <0.001 |
| Rural (%) | 874 (20.0) | 189 (15.1) | 205 (18.0) | 160 (15.6) | 11 (11.6) | 35 (8.9) | 51 (11.1) | <0.001 |
| Body mass index, kg/m^2^ | 23.7±3.8 | 23.2±3.9 | 24.5±3.3 | 25.2±3.5 | 22.5±3.2 | 19.1±1.6 | 18.9±2.1 | <0.001 |
| MMSE | 16.0 [13.0, 19.0] | 14.0 [11.0, 17.0] | 15.0 [12.0, 18.0] | 13.0 [10.0, 16.0] | 13.0 [7.0, 16.0] | 14.0 [10.0, 17.0] | 11.0 [8.0, 15.0] | <0.001 |
| Smoking status (%) |  |  |  |  |  |  |  | <0.001 |
| Never smoker | 1449 (33.2) | 370 (29.6) | 312 (27.4) | 245 (24.0) | 30 (31.6) | 119 (30.6) | 139 (30.5) |  |
| Past smoker | 381 (8.7) | 103 (8.2) | 88 (7.7) | 101 (9.9) | 5 (5.3) | 29 (7.5) | 38 (8.3) |  |
| Current smoker | 2530 (58.0) | 776 (62.1) | 739 (64.9) | 676 (66.1) | 60 (63.2) | 241 (62.0) | 279 (61.2) |  |
| Drinking status, % |  |  |  |  |  |  |  | <0.001 |
| Never drinker | 1639 (37.6) | 384 (30.7) | 331 (29.0) | 231 (22.6) | 19 (20.0) | 123 (31.4) | 109 (23.6) |  |
| Past drinker | 332 (7.6) | 88 (7.0) | 90 (7.9) | 107 (10.4) | 12 (12.6) | 36 (9.2) | 57 (12.4) |  |
| Current drinker | 2391 (54.8) | 780 (62.3) | 722 (63.2) | 686 (67.0) | 64 (67.4) | 233 (59.4) | 295 (64.0) |  |
| Ideal physical activity, % | 3420 (78.3) | 875 (69.9) | 781 (68.3) | 583 (56.8) | 48 (50.5) | 278 (70.6) | 242 (52.5) | <0.001 |
| Optimal sleep duration, % | 2913 (66.7) | 742 (59.3) | 727 (63.6) | 580 (56.5) | 44 (46.3) | 177 (44.9) | 192 (41.6) | <0.001 |
| Chronic diseases |  |  |  |  |  |  |  |  |
| Hypertension, % | 1515 (34.7) | 523 (41.8) | 448 (39.2) | 614 (59.8) | 43 (45.3) | 136 (34.5) | 208 (45.1) | <0.001 |
| Diabetes, % | 3124 (71.5) | 879 (70.2) | 844 (73.8) | 769 (74.9) | 67 (70.5) | 280 (71.1) | 321 (69.6) | 0.120 |
| Chronic lung disease, % | 346 (7.9) | 138 (11.0) | 105 (9.2) | 125 (12.2) | 13 (13.7) | 54 (13.7) | 86 (18.7) | <0.001 |
| Asthma, % | 153 (3.5) | 73 (5.8) | 43 (3.8) | 64 (6.2) | 6 (6.3) | 24 (6.1) | 38 (8.2) | <0.001 |
| Cancer, % | 33 (0.8) | 9 (0.7) | 11 (1.0) | 13 (1.3) | 0 (0.0) | 2 (0.5) | 3 (0.7) | 0.592 |
| Blood pressure  lowering medication, % | 639 (14.6) | 226 (18.1) | 234 (20.5) | 365 (35.5) | 23 (24.2) | 45 (11.4) | 79 (17.1) | <0.001 |
| Blood glucose  lowering medication, % | 135 (3.1) | 53 (4.2) | 60 (5.2) | 64 (6.2) | 6 (6.3) | 8 (2.0) | 7 (1.5) | <0.001 |
| Solid fuel for cooking, % | 2360 (54.0) | 790 (63.1) | 719 (62.9) | 690 (67.2) | 73 (76.8) | 288 (73.1) | 341 (74.0) | <0.001 |
| Solid fuel for heating, % | 3345 (76.6) | 1026 (81.9) | 958 (83.8) | 868 (84.5) | 89 (93.7) | 352 (89.3) | 410 (88.9) | <0.001 |
| Handgrip strength | 35.0 [29.0, 42.5] | 31.5 [26.0, 38.50] | 29.6 [24.0, 37.0] | 26.5 [20.5, 34.0] | 25.0 [20.7, 30.6] | 25.5 [20.0, 32.0] | 22.3 [17.5, 27.0] | <0.001 |
| Appendicular skeletal muscle | 17.8 [14.3, 20.8] | 16.5 [13.5, 19.8] | 16.7 [14.2, 20.1] | 16.6 [14.0, 20.0] | 15.6 [12.0, 18.6] | 12.2 [10.6, 17.3] | 11.6 [10.0, 16.2] | <0.001 |

MMSE, Mini-Mental State Examination,

**Table S2.** Associations between sarcopenia transitions and risk of cardiovascular diseases

| **Sarcopenia transition** | **Hazard ratio (95%CI)** | | |
| --- | --- | --- | --- |
|  | **Cardiovascular disease** | **Heart disease** | **Stroke** |
| Stable normal | 1.00 (reference) | 1.00 (reference) | 1.00 (reference) |
| Normal to possible/confirmed sarcopenia | 1.40 (1.17-1.66) | 1.44 (1.18-1.75) | 1.20 (0.86-1.67) |
| Possible sarcopenia to normal | 1.13 (0.94-1.36) | 1.08 (0.87-1.34) | 1.21 (0.87-1.67) |
| Stable possible sarcopenia | 1.71 (1.43-2.05) | 1.59 (1.29-1.96) | 2.04 (1.51-2.76) |
| Possible to confirmed sarcopenia | 1.73 (1.06-2.83) | 1.73 (1.03-2.44) | 1.54 (0.62-3.81) |
| Confirmed sarcopenia to normal/possible sarcopenia | 1.42 (1.05-1.90) | 1.34 (1.01-1.73) | 1.53 (0.92-2.55) |
| Stable confirmed sarcopenia | 1.55 (1.16-2.06) | 1.62 (1.17-2.23) | 1.34 (0.78-2.28) |

**Table S3.** Associations between sarcopenia transitions and risk of cardiovascular diseases stratified by sex

| **Sarcopenia transition** | **Male** | **Female** | **P for interaction** |
| --- | --- | --- | --- |
|  | **HR (95% CI)** | **HR (95% CI)** |  |
| Stable normal | 1.00 (reference) | 1.00 (reference) |  |
| Normal to possible/confirmed sarcopenia | 1.35 (1.06-1.72) | 1.45 (1.12-1.87) | 0.744 |
| Possible sarcopenia to normal | 1.43 (0.96-2.11) | 0.92 (0.67-1.25) | 0.064 |
| Stable possible sarcopenia | 1.81 (1.43-2.28) | 1.62 (1.22-2.16) | 0.534 |
| Possible to confirmed sarcopenia | 2.36 (1.26-4.42) | 1.10 (0.48-2.50) | 0.012 |
| Confirmed sarcopenia to normal/possible sarcopenia | 1.32 (1.04-1.66) | 1.40 (0.89-2.19) | 0.069 |
| Stable confirmed sarcopenia | 1.65 (1.15-2.37) | 1.39 (0.85-2.27) | 0.056 |

**Table S4.** Associations between sarcopenia transitions and risk of cardiovascular diseases stratified by age group

| **Sarcopenia transition** | **Middle aged** | **Elderly** | **P for interaction** |
| --- | --- | --- | --- |
|  | **HR (95%CI)** | **HR (95%CI)** |  |
| Stable normal | 1.00 (reference) | 1.00 (reference) |  |
| Normal to possible/confirmed sarcopenia | 1.33 (1.08-1.65) | 1.51 (1.09-2.10) | 0.744 |
| Possible sarcopenia to normal | 1.12 (0.91-1.38) | 1.20 (0.78-1.83) | 0.779 |
| Stable possible sarcopenia | 1.89 (1.53-2.33) | 1.48 (1.05-2.07) | 0.200 |
| Possible to confirmed sarcopenia | 1.91 (0.79-4.65) | 1.85 (1.00-3.44) | 0.662 |
| Confirmed sarcopenia to normal/possible sarcopenia | 1.31 (0.83-2.07) | 1.40 (0.92-2.12) | 0.787 |
| Stable confirmed sarcopenia | 1.94 (1.18-3.17) | 1.45 (0.98-2.15) | 0.167 |

**Table S5.** Associations between sarcopenia transitions and risk of cardiovascular diseases stratified by smoking status

| **Sarcopenia transition** | **Never smoker** | **Former smoker** | **Current smoker** | **P for interaction** |
| --- | --- | --- | --- | --- |
|  | **HR (95% CI)** | **HR (95% CI)** | **HR (95% CI)** |  |
| Stable normal | 1.00 (reference) | 1.00 (reference) | 1.00 (reference) |  |
| Normal to possible/confirmed sarcopenia | 1.55 (1.13-2.15) | 1.04 (0.58-1.87) | 1.39 (1.11-1.74) | 0.269 |
| Possible sarcopenia to normal | 0.95 (0.65-1.40) | 1.03 (0.55-1.92) | 1.22 (0.97-1.53) | 0.068 |
| Stable possible sarcopenia | 1.73 (1.21-2.49) | 2.01 (1.12-3.60) | 1.68 (1.35-2.11) | 0.740 |
| Possible to confirmed sarcopenia | 1.89 (0.83-4.33) | / | 1.77 (0.95-3.30) | 0.507 |
| Confirmed sarcopenia to normal/possible sarcopenia | 1.64 (0.97-2.78) | 2.14 (0.76-5.99) | 1.31 (0.89-1.91) | 0.385 |
| Stable confirmed sarcopenia | 1.68 (0.98-2.88) | 1.57 (0.50-4.92) | 1.51 (1.05-2.17) | 0.505 |

**Table S6.** Associations between sarcopenia transitions and risk of cardiovascular diseases stratified by physical activity status

| **Sarcopenia transition** | **Physically inactive** | **Physically active** | **P for interaction** |
| --- | --- | --- | --- |
|  | **HR (95% CI)** | **HR (95% CI)** |  |
| Stable normal | 1.00 (reference) | 1.00 (reference) |  |
| Normal to possible/confirmed sarcopenia | 1.11 (0.80-1.54) | 1.51 (1.23-1.86) | 0.428 |
| Possible sarcopenia to normal | 0.77 (0.54-1.10) | 1.30 (1.05-1.61) | 0.018 |
| Stable possible sarcopenia | 1.12 (0.82-1.53) | 2.10 (1.69-2.61) | 0.013 |
| Possible to confirmed sarcopenia | 0.81 (0.35-1.87) | 2.96 (1.61-5.44) | 0.007 |
| Confirmed sarcopenia to normal/possible sarcopenia | 0.91 (0.51-1.60) | 1.66 (1.17-2.35) | 0.142 |
| Stable confirmed sarcopenia | 0.91 (0.55-1.49) | 2.01 (1.42-2.86) | 0.014 |

**Table S7.** Associations between sarcopenia transitions and risk of cardiovascular diseases stratified by diabetes status

| **Sarcopenia transition** | **Without diabetes** | **With diabetes** | **P for interaction** |
| --- | --- | --- | --- |
|  | **HR (95% CI)** | **HR (95% CI)** |  |
| Stable normal | 1.00 (reference) | 1.00 (reference) |  |
| Normal to possible/confirmed sarcopenia | 1.57 (1.11-2.23) | 1.32 (1.08-1.62) | 0.273 |
| Possible sarcopenia to normal | 1.20 (0.82-1.77) | 1.12 (0.91-1.39) | 0.797 |
| Stable possible sarcopenia | 2.14 (1.48-3.10) | 1.65 (1.35-2.03) | 0.179 |
| Possible to confirmed sarcopenia | 4.13 (1.82-9.34) | 1.16 (0.61-2.20) | 0.014 |
| Confirmed sarcopenia to normal/possible sarcopenia | 2.28 (1.33-3.90) | 1.04 (0.74-1.47) | 0.027 |
| Stable confirmed sarcopenia | 1.77 (0.97-3.24) | 1.30 (0.95-1.80) | 0.747 |

**Table S8.** Associations between sarcopenia transitions and cardiovascular diseases of different baseline sarcopenia status stratified by sex

| **Sarcopenia transition** | **Female** | **Male** | **P for interaction** |
| --- | --- | --- | --- |
|  | **HR (95%CI)** | **HR (95%CI)** |  |
| Stable normal | 1.00 (reference) | 1.00 (reference) |  |
| Normal to possible/confirmed sarcopenia | 1.30 (0.96-1.75) | 1.62 (1.19-2.22) | 0.440 |
| Possible sarcopenia to normal | 1.00 (reference) | 1.00 (reference) |  |
| Stable possible sarcopenia | 0.88 (0.63-1.23) | 0.47 (0.29-0.76) | 0.016 |
| Possible to confirmed sarcopenia | 1.70 (1.01-3.33) | 0.42 (0.13-1.38) | 0.010 |
| Confirmed sarcopenia to normal/possible sarcopenia | 1.00 (reference) | 1.00 (reference) |  |
| Stable confirmed sarcopenia | 0.78 (0.44-1.38) | 0.69 (0.32-1.47) | 0.508 |

| **Sarcopenia transition** | **Middle aged** | **Elderly** | **P for interaction** |
| --- | --- | --- | --- |
|  | **HR (95%CI)** | **HR (95%CI)** |  |
| Stable normal | 1.00 (reference) | 1.00 (reference) |  |
| Normal to possible/confirmed sarcopenia | 1.44 (1.11-1.87) | 1.41 (0.96-2.09) | 0.068 |
| Stable possible sarcopenia | 1.00 (reference) | 1.00 (reference) |  |
| Possible sarcopenia to normal | 0.66 (0.48-0.91) | 0.84 (0.50-1.41) | 0.138 |
| Possible to confirmed sarcopenia | 2.18 (0.84-5.68) | 0.82 (0.39-1.72) | 0.288 |
| Confirmed sarcopenia to normal/possible sarcopenia | 1.00 (reference) | 1.00 (reference) |  |
| Stable confirmed sarcopenia | 0.83 (0.35-1.98) | 0.91 (0.54-1.52) | 0.373 |

**Table S9.** Associations between sarcopenia transitions and cardiovascular diseases of different baseline sarcopenia status stratified by age group

**Table S10.** Associations between sarcopenia transitions and cardiovascular diseases of different baseline sarcopenia status stratified by smoking status

| **Sarcopenia transition** | **Never smoker** | **Past smoker** | **Current smoker** | **P for interaction** |
| --- | --- | --- | --- | --- |
|  | **HR (95%CI)** | **HR (95%CI)** | **HR (95%CI)** |  |
| Stable normal | 1.00 (reference) | 1.00 (reference) | 1.00 (reference) |  |
| Normal to possible/confirmed sarcopenia | 1.92 (1.30-2.84) | 0.85 (0.39-1.86) | 1.36 (1.03-1.80) | 0.210 |
| Stable possible sarcopenia | 1.00 (reference) |  |  |  |
| Possible sarcopenia to normal | 0.43 (0.23-0.81) | 0.59 (0.24-1.49) | 0.88 (0.63-1.24) | 0.123 |
| Possible to confirmed sarcopenia | 1.72 (0.62-4.78) | / | 1.14 (0.55-2.36) | 0.569 |
| Confirmed sarcopenia to normal/possible sarcopenia | 1.00 (reference) | 1.00 (reference) | 1.00 (reference) |  |
| Stable confirmed sarcopenia | 1.02 (0.42-2.49) |  | 0.76 (0.43-1.34) | 0.636 |

**Table S11.** Associations between sarcopenia transitions and cardiovascular diseases of different baseline sarcopenia status stratified by physical activity

| **Sarcopenia transition** | **Physically inactive** | **Physically active** | **P for interaction** |
| --- | --- | --- | --- |
|  | **HR (95%CI)** | **HR (95%CI)** |  |
| Stable normal | 1.00 (reference) | 1.00 (reference) |  |
| Normal to possible/confirmed sarcopenia | 1.37 (0.90-2.07) | 1.49 (1.15-1.93) | 0.317 |
| Stable possible sarcopenia | 1.00 (reference) | 1.00 (reference) |  |
| Possible sarcopenia to normal | 0.81 (0.52-1.25) | 0.63 (0.45-0.89) | 0.046 |
| Possible to confirmed sarcopenia | 1.14 (0.54-2.41) | 0.98 (0.39-2.47) | 0.167 |
| Confirmed sarcopenia to normal/possible sarcopenia | 1.00 (reference) | 1.00 (reference) |  |
| Stable confirmed sarcopenia | 0.48 (0.20-1.15) | 0.94 (0.55-1.59) | 0.878 |

**Table S12.** Associations between sarcopenia transitions and cardiovascular diseases of different baseline sarcopenia status stratified by diabetes

| **Sarcopenia transition** | **No diabetes** | **Diabetes** | **P for interaction** |
| --- | --- | --- | --- |
|  | **HR (95%CI)** | **HR (95%CI)** |  |
| Stable normal | 1.00 (reference) | 1.00 (reference) |  |
| Normal to possible/confirmed sarcopenia | 1.35 (1.05-1.73) | 1.85 (1.19-2.88) | 0.251 |
| Stable possible sarcopenia | 1.00 (reference) | 1.00 (reference) |  |
| Possible sarcopenia to normal | 0.74 (0.54-0.99) | 0.61 (0.35-1.06) | 0.378 |
| Possible to confirmed sarcopenia | 0.70 (0.32-1.53) | 2.39 (0.91-6.25) | 0.126 |
| Confirmed sarcopenia to normal/possible sarcopenia | 1.00 (reference) | 1.00 (reference) |  |
| Stable confirmed sarcopenia | 0.69 (0.41-1.14) | 1.28 (0.45-3.69) | 0.161 |

**Table S13.** Associations between types of indoor fuel use and sarcopenia transitions stratified by sex

| **Fuel type** |  | **Normal to possible/confirmed sarcopenia** | | | | **Possible sarcopenia to normal** | | | | **Confirmed sarcopenia to normal/possible sarcopenia** | | | |
| --- | --- | --- | --- | --- | --- | --- | --- | --- | --- | --- | --- | --- | --- |
|  |  | **Odds ratio (95%CI)** | | ***p* value** | ***p* for interaction** | **Odds ratio (95%CI)** | | ***p* value** | ***p* for interaction** | **Odds ratio (95%CI)** | | ***p* value** | ***p* for interaction** |
|  |  | **Clean fuel** | **Solid fuel** |  |  | **Solid fuel** | **Clean fuel** |  |  | **Solid fuel** | **Clean fuel** |  |  |
| Cooking | Female | ref | 1.38 (1.06-1.80) | 0.016 | **0.015** | ref | 1.25 (0.90-1.75) | 0.185 | 0.740 | ref | 0.88 (0.51-1.51) | 0.632 | 0.155 |
|  | Male | ref | 0.96 (0.73-1.26) | 0.757 |  | ref | 1.51 (0.98-2.34) | 0.066 |  | ref | 1.60 (0.78-3.33) | 0.199 |  |
| Heating | Female | ref | 1.36 (1.09-1.89) | 0.049 | 0.062 | ref | 1.06 (0.70-1.62) | 0.776 | 0.718 | ref | 0.92 (0.39-2.15) | 0.851 | 0.456 |
|  | Male | ref | 1.16 (0.83-1.63) | 0.382 |  | ref | 0.87 (0.49-1.56) | 0.636 |  | ref | 1.26 (0.50-3.17) | 0.618 |  |

**Table S14.** Associations between types of indoor fuel use and sarcopenia transitions stratified by age group

| **Fuel type** |  | **Normal to possible/confirmed sarcopenia** | | | | **Possible sarcopenia to normal** | | | | **Confirmed sarcopenia to normal/possible sarcopenia** | | | |
| --- | --- | --- | --- | --- | --- | --- | --- | --- | --- | --- | --- | --- | --- |
|  |  | **Odds ratio (95%CI)** | | ***p* value** | ***p* for interaction** | **Odds ratio (95%CI)** | | ***p* value** | ***p* for interaction** | **Odds ratio (95%CI)** | | ***p* value** | ***p* for interaction** |
|  |  | **Clean fuel** | **Solid fuel** |  |  | **Solid fuel** | **Clean fuel** |  |  | **Solid fuel** | **Clean fuel** |  |  |
| Cooking | Middle aged | ref | **1.27 (1.02-1.59)** | 0.035 | 0.059 | ref | 1.36 (0.99-1.87) | 0.062 | 0.495 | ref | 2.13 (0.89-3.50) | 0.100 | 0.132 |
|  | Elderly | ref | 1.36 (0.99-1.89) | 0.762 |  | ref | 1.17 (0.72-1.88) | 0.526 |  | ref | 0.82 (0.48-1.36) | 0.441 |  |
| Heating | Middle aged | ref | 1.05 (0.66-1.36) | 0.104 | 0.462 | ref | 1.17 (0.72-1.88) | 0.526 | 0.151 | ref | 1.66 (0.33-2.49) | 0.553 | 0.380 |
|  | Elderly | ref | 1.16(0.83-0.63) | 0.302 |  | ref | 1.19 (0.78-1.81) | 0.423 |  | ref | 1.10 (0.56-2.13) | 0.773 |  |

**Table S15.** Associations between types of indoor fuel use and sarcopenia transitions stratified by smoking status

| **Fuel type** |  | **Normal to possible/confirmed sarcopenia** | | | | **Possible sarcopenia to normal** | | | | **Confirmed sarcopenia to normal/possible sarcopenia** | | | |
| --- | --- | --- | --- | --- | --- | --- | --- | --- | --- | --- | --- | --- | --- |
|  |  | **Odds ratio (95%CI)** | | ***p* value** | ***p* for interaction** | **Odds ratio (95%CI)** | | ***p* value** | ***p* for interaction** | **Odds ratio (95%CI)** | | ***p* value** | ***p* for interaction** |
|  |  | **Clean fuel** | **Solid fuel** |  |  | **Solid fuel** | **Clean fuel** |  |  | **Solid fuel** | **Clean fuel** |  |  |
| Cooking | Never  smoker | ref | 1.04 (0.74-1.47) | 0.081 | 0.292 | ref | 1.27 (0.73-2.25) | 0.203 | 0.981 | ref | 0.97 (0.42-2.23) | 0.639 | 0.757 |
|  | Past smoker | ref | 1.96 (0.94-2.33) | 0.826 |  | ref | 1.97 (0.71-5.79) | 0.401 |  | ref | 2.17 (0.08-7.86) | 0.944 |  |
|  | Current  smoker | ref | 1.17 (0.92-1.48) | 0.205 |  | ref | 1.23 (0.89-1.70) | 0.201 |  | ref | 1.08 (0.84-1.84) | 0.770 |  |
| Heating | Never  smoker | ref | 1.43 (0.92-2.27) | 0.281 | 0.668 | ref | 1.14 (0.53-2.51) | 0.323 | 0.671 | ref | 1.26 (0.38-4.26) | 0.367 | 0.860 |
|  | Past smoker | ref | 0.63 (0.28-1.47) | 0.123 |  | ref | 0.93 (0.64-1.86) | 0.736 |  | ref | 5.84 (0.06-7.46) | 0.702 |  |
|  | Current  smoker | ref | 1.29 (0.97-1.73) | 0.082 |  | ref | 1.04 (0.69-1.57) | 0.832 |  | ref | 1.06 (0.50-2.22) | 0.876 |  |

**Table S16.** Associations between types of indoor fuel use and sarcopenia transitions stratified by physical activity status

| **Fuel type** |  | **Normal to possible/confirmed sarcopenia** | | | | **Possible sarcopenia to normal** | | | | **Confirmed sarcopenia to normal/possible sarcopenia** | | | |
| --- | --- | --- | --- | --- | --- | --- | --- | --- | --- | --- | --- | --- | --- |
|  |  | **Odds ratio (95%CI)** | | ***p* value** | ***p* for interaction** | **Odds ratio (95%CI)** | | ***p* value** | ***p* for interaction** | **Odds ratio (95%CI)** | | ***p* value** | ***p* for interaction** |
|  |  | **Clean fuel** | **Solid fuel** |  |  | **Solid fuel** | **Clean fuel** |  |  | **Solid fuel** | **Clean fuel** |  |  |
| Cooking | Physically inactive | ref | 1.27 (0.90-1.80) | 0.171 | 0.0519 | ref | 1.19 (0.79-1.77) | 0.402 | 0.177 | ref | 0.57 (0.28-1.12) | 0.110 | 0.078 |
|  | Physically active | ref | 1.13 (1.02-1.42) | 0.025 |  | ref | 1.48 (1.03-2.13) | 0.033 |  | ref | 1.71 (0.95-3.17) | 0.078 |  |
| Heating | Physically inactive | ref | 1.26 (0.87-1.84) | 0.224 | 0.876 | ref | 1.01 (0.56-1.47) | 0.708 | 0.306 | ref | 1.74 (0.69-4.31) | 0.231 | 0.689 |
|  | Physically active | ref | 1.28 (0.95-1.73) | 0.111 |  | ref | 1.18 (0.72-1.94) | 0.519 |  | ref | 0.89 (0.39-2.00) | 0.775 |  |

**Table S17.** Associations between types of indoor fuel use and sarcopenia transitions stratified by diabetes status

| **Fuel type** |  | **Normal to possible/confirmed sarcopenia** | | | | **Possible sarcopenia to normal** | | | | **Confirmed sarcopenia to normal/possible sarcopenia** | | | |
| --- | --- | --- | --- | --- | --- | --- | --- | --- | --- | --- | --- | --- | --- |
|  |  | **Odds ratio (95%CI)** | | ***p* value** | ***p* for interaction** | **Odds ratio (95%CI)** | | ***p* value** | ***p* for interaction** | **Odds ratio (95%CI)** | | ***p* value** | ***p* for interaction** |
|  |  | **Clean fuel** | **Solid fuel** |  |  | **Solid fuel** | **Clean fuel** |  |  | **Solid fuel** | **Clean fuel** |  |  |
| Cooking | Without diabetes | ref | 1.22 (0.98-1.53) | 0.083 | 0.806 | ref | 1.22 (0.90-1.67) | 0.201 | 0.826 | ref | 1.09 (0.48-1.30) | 0.356 | **0.040** |
|  | With  diabetes | ref | 1.06 (0.75-1.51) | 0.747 |  | ref | 1.60 (0.94-2.75) | 0.085 |  | ref | 2.88 (1.12-7.90) | 0.032 |  |
| Heating | Without diabetes | ref | 1.31 (1.00-1.73) | 0.051 | 0.885 | ref | 1.07 (0.73-1.59) | 0.724 | 0.885 | ref | 1.36 (0.70-2.64) | 0.364 | 0.937 |
|  | With  diabetes | ref | 1.20 (0.78-1.90) | 0.416 |  | ref | 0.87 (0.43-1.76) | 0.689 |  | ref | 0.78 (0.19-3.12) | 0.725 |  |

**Table S18: Missing rates of included covariates**

| **Variables** | **Number of missing values** | **Percentage of missing values** |
| --- | --- | --- |
| Physical performance at 2011 | 362 | 4.14% |
| Physical performance at 2013 | 309 | 3.54% |
| HGS at 2013 | 114 | 1.30% |
| HGS at 2011 | 97 | 1.11% |
| ASM at 2011 | 38 | 0.43% |
| ASM at 2013 | 22 | 0.25% |
| Baseline age | 0 | / |
| Sex | 0 | / |
| Information on residence (urban/rural) | 5 | 0.06% |
| Information on income | 304 | 3.48% |
| Information on MMSE | 203 | 2.32% |
| Information on education | 0 | / |
| Information on marital status | 0 | 0.00% |
| Body mass index | 0 | 0.00% |
| Information on smoking | 31 | 0.35% |
| Information on drinking | 29 | 0.33% |
| Information on physical activity | 0 | 0.00% |
| Information on sleep | 0 | 0.00% |
| History of diabetes | 0 | 0.00% |
| History of hypertension | 0 | 0.00% |
| History of heart | 0 | 0.00% |
| History of stroke | 0 | 0.00% |
| History of lung | 0 | 0.00% |
| History of asthma | 0 | 0.00% |
| History of cancer | 0 | 0.00% |
| Information on cooking fuel | 0 | 0.00% |
| Information on heating fuel | 0 | 0.00% |

**Table S19.** Associations between sarcopenia transitions and risk of cardiovascular diseases (missing covariates deleted)

| **Sarcopenia transition** | **Hazard ratio (95%CI)** | | |
| --- | --- | --- | --- |
|  | **Cardiovascular disease** | **Heart disease** | **Stroke** |
| Stable normal | 1.00 (reference) | 1.00 (reference) | 1.00 (reference) |
| Normal to possible/confirmed sarcopenia | 1.43 (1.20-1.70) | 1.48 (1.21-1.81) | 1.20 (0.86-1.69) |
| Possible sarcopenia to normal | 1.14 (0.95-1.37) | 1.10 (0.89-1.37) | 1.20 (0.86-1.68) |
| Stable possible sarcopenia | 1.71 (1.42-2.05) | 1.59 (1.29-1.97) | 1.99 (1.46-2.72) |
| Possible to confirmed sarcopenia | 1.69 (1.10-2.29) | 1.76 (1.10-2.41) | 1.33 (0.49-3.67) |
| Confirmed sarcopenia to normal/possible sarcopenia | 1.47 (1.09-1.98) | 1.69 (1.06-2.70) | 1.65 (0.99-2.76) |
| Stable confirmed sarcopenia | 1.61 (1.20-2.15) | 1.68 (1.21-2.33) | 1.40 (0.81-2.42) |

**Table S20.** Associations between sarcopenia transitions and cardiovascular diseases of different baseline sarcopenia status (missing covariates deleted)

| **Sarcopenia transition** | **Hazard ratio (95%CI)** | | |
| --- | --- | --- | --- |
|  | **Cardiovascular disease** | **Heart disease** | **Stroke** |
| Stable normal | 1.00 (reference) | 1.00 (reference) | 1.00 (reference) |
| Normal to possible/confirmed sarcopenia | 1.47 (1.15-1.89) | 1.50 (1.13-2.00) | 1.42 (0.88-2.29) |
| Stable possible sarcopenia | 1.00 (reference) | 1.00 (reference) | 1.00 (reference) |
| Possible sarcopenia to normal | 0.65 (0.46-0.90) | 0.69 (0.47-1.01) | 0.54 (0.30-0.96) |
| Possible to confirmed sarcopenia | 1.24 (0.59-2.64) | 1.49 (0.65-3.38) | 1.26 (0.18-3.43) |
| Stable confirmed sarcopenia | 1.00 (reference) | 1.00 (reference) | 1.00 (reference) |
| Confirmed sarcopenia to normal/possible | 0.97 (0.56-1.91) | 0.93 (0.48-1.80) | 1.68 (0.43-2.60) |

**Table S21.** Associations between types of indoor fuel use and sarcopenia transitions (missing covariates deleted)

| **Fuel type** | **Normal to possible/confirmed sarcopenia ^*^** | | | **Possible sarcopenia to normal ^#^** | | | **Confirmed sarcopenia to normal/possible ^$^** | | |
| --- | --- | --- | --- | --- | --- | --- | --- | --- | --- |
|  | **OR (95% CI)** | | ***p* value** | **OR (95% CI)** | | ***p* value** | **OR (95% CI)** | | ***p* value** |
|  | **Clean fuel** | **Solid fuel** |  | **Solid fuel** | **Clean fuel** |  | **Solid fuel** | **Clean fuel** |  |
| Cooking | 1.00 (reference) | 1.33 (1.14, 1.55) | <.001 | 1.00 (reference) | 1.31 (1.05, 1.64) | 0.016 | 1.00 (reference) | 1.12 (0.74, 1.66) | 0.592 |
| Heating | 1.00 (reference) | 1.22 (1.01, 1.46) | 0.036 | 1.00 (reference) | 1.00 (0.75, 1.33) | 0.998 | 1.00 (reference) | 1.03 (0.60, 1.77) | 0.919 |
